# Patient‐derived organoids for personalized gallbladder cancer modelling and drug screening

**DOI:** 10.1002/ctm2.678

**Published:** 2022-01-24

**Authors:** Bo Yuan, Xiaofang Zhao, Xiang Wang, Erdong Liu, Chunliang Liu, Yali Zong, Youhai Jiang, Minghui Hou, Yao Chen, Lei Chen, Yongjie Zhang, Hongyang Wang, Jing Fu

**Affiliations:** ^1^ International Cooperation Laboratory on Signal Transduction Ministry of Education Key Laboratory on Signaling Regulation and Targeting Therapy of Liver Cancer Shanghai Key Laboratory of Hepato‐biliary Tumor Biology Eastern Hepatobiliary Surgery Hospital Second Military Medical University Shanghai China; ^2^ Research Center for Organoids The First Affiliated Hospital of Zhengzhou University Zhengzhou Henan China; ^3^ Second Department of Biliary Surgery Eastern Hepatobiliary Surgery Hospital Second Military Medical University Shanghai China; ^4^ School of Life Sciences Fudan University Shanghai China; ^5^ Division of Life Sciences and Medicine Cancer Research Center The First Affiliated Hospital of USTC University of Science and Technology of China Hefei Anhui China

**Keywords:** drug screening, gallbladder cancer, HDAC, organoids

## Abstract

**Background:**

Gallbladder carcinoma (GBC) is a relatively rare but highly aggressive cancer with late clinical detection and a poor prognosis. However, the lack of models with features consistent with human gallbladder tumours has hindered progress in pathogenic mechanisms and therapies.

**Methods:**

We established organoid lines derived from human GBC as well as normal gallbladder and benign gallbladder adenoma (GBA) tissues. The histopathology signatures of organoid cultures were identified by H&E staining, immunohistochemistry and immunofluorescence. The genetic and transcriptional features of organoids were analysed by whole‐exome sequencing and RNA sequencing. A set of compounds targeting the most active signalling pathways in GBCs were screened for their ability to suppress GBC organoids. The antitumour effects of candidate compounds, CUDC‐101 and CUDC‐907, were evaluated in vitro and in vivo.

**Results:**

The established organoids were cultured stably for more than 6 months and closely recapitulated the histopathology, genetic and transcriptional features, and intratumour heterogeneity of the primary tissues at the single‐cell level. Notably, expression profiling analysis of the organoids revealed a set of genes that varied across the three subtypes and thus may participate in the malignant progression of gallbladder diseases. More importantly, we found that the dual PI3K/HDAC inhibitor CUDC‐907 significantly restrained the growth of various GBC organoids with minimal toxicity to normal gallbladder organoids.

**Conclusions:**

Patient‐derived organoids are potentially a useful platform to explore molecular pathogenesis of gallbladder tumours and discover personalized drugs.

## Graphical abstract

We successfully established organoid lines derived from human GBC that recapitulated the histopathology, genetic and transcriptional features of the primary tissues. In drug screening experiment, we found that the dual PI3K/HDAC inhibitor CUDC‐907 significantly restrained the growth of various GBC organoids with minimal toxicity to normal gallbladder organoids.

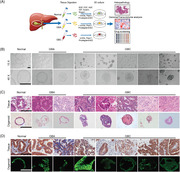

## 1 BACKGROUND

Gallbladder carcinoma (GBC) is the most common type of the biliary tract cancers and ranking as the sixth most common gastrointestinal malignancy.^1^, ^2^ The incidence of GBC shows regional variations worldwide and is infrequent in developed countries but common in some specific geographical regions of developing countries. The overall prognosis of GBC is very poor. Surgical resection is the only method that can cure patients with localized GBC, but the recurrence rate can be as high as 65%.^3^ Adjuvant therapy strategies, including chemotherapy, radiotherapy or combination treatment, are used to improve the outcomes of patients with advanced GBC. However, in many patients with this highly heterogeneous tumour, the therapeutic response is not satisfactory. There is an urgent need to develop more personalized and targeted therapies.^4^, ^5^, ^6^ One of the major barriers to the development of novel treatments is the lack of appropriate models that can accurately recapitulate the histological complexity and genetic heterogeneity of human cancers.

Organoids, recently developed three‐dimensional (3D) culture technologies, faithfully recapitulate the architecture and function of primary tissues.^7^ Patient‐derived organoids (PDOs) derived from individual tumour patients can indefinitely expand and accurately recapitulate the morphological and molecular features of the original tumour. To date, tumour organoids have been established for a wide range of cancer types, such as colon, pancreatic, liver and prostate tumours.^8^, ^9^, ^10^, ^11^ These PDOs were subsequently employed for drug screening and tailored treatment in cancer therapy. Increasing evidences demonstrated that PDOs can accurately predict patient response to anticancer treatment.^12^, ^13^, ^14^, ^15^ Here, by using organoid culture technology, we established organoids derived from human normal gallbladder, benign gallbladder adenoma (GBA) and GBC tissues. The morphology, genetic characteristics, transcriptional profiles and intratumour heterogeneity of the organoids were analysed. Finally, we screened a series of compounds to identify drugs that could effectively suppress growth of GBC organoids.

## 2 MATERIALS AND METHODS

### 2.1 Human specimens

We obtained human tissues through surgeries performed at the Eastern Hepatobiliary Surgery Hospital, Shanghai, China. The tumour samples were processed for histology, RNA and DNA isolation, or dissociated for organoid culture. Normal gallbladder tissues were collected through gallstone surgery. GBC samples were obtained from patients who underwent radical resection in Eastern Hepatobiliary Surgery Hospital from Jan 2016 to Nov 2019. All patients’ diagnoses were histologically confirmed. We excluded cases with recurrent GBC or those receiving neoadjuvant treatment. This cohort was followed up until Jul 2020, with a follow‐up from 7.2 to 54.4 months. The age was 39–84 years (median 64 years). The detailed clinicopathologic features of 100 GBC patients were shown in Table S3. The variables we collected included gender, age, anti‐HBc status, gallstone, gallbladder polyps, CEA level, CA 19‐9 level, location, type of surgery, surgical margin, invasion (liver, vascular, bile duct), metastasis (lymph node, distance), TNM stage, tumour differentiation and histology type. This study was approved by the Ethical Committee of the Second Military Medical University, and informed consent was signed by each participant.

### 2.2 Establishment of organoids

Patient‐derived specimen was minced and incubated in digestion solution (2.5 mg/ml Collagenase IV, Roche) for 30–60 min at 37°C. After stopping digestion by dulbecco's modified eagle medium (DMEM) with 10% fetal bovine serum (FBS), the suspension was filtered through a cell strainer (100 μm) and centrifuged at 1000 rpm for 5 min. The pellet was washed using cold Advanced DMEM/F12 (GIBCO, USA), and then mixed with matrigel (BD Transduction Laboratories, USA). After counting, cells were seeded and cultured in six‐multiwell suspension plate (10,000–20,000 cells per well). The organoid culture medium for normal and GBA samples consisted of Advanced DMEM/F12 medium, 1:50 B27 supplement, 1:100 N2 supplement, 1.25 mM *N*‐acetyl‐l‐cysteine, 250 ng/ml Rspo‐1, 100 ng/ml Wnt3a, 100 ng/ml Noggin, 50 ng/ml EGF, 100 ng/ml FGF10, 100 ng/ml IGF, 25 ng/ml recombinant human HGF, 10 nM gastrin, 10 mM Y27632, 10 mM nicotinamide, 5 μM A8301, 10 μM forskolin, 5 μg/ml Prostaglandin E2, 3 nM dexamethasone, 1:500 Primocin, 1% penicillin/streptomycin, 1% Glutamax and 1% hydroxyethyl piperazineethanesulfonic acid (HEPES). GBC samples were cultured in the above media without Wnt3a, Rspo1 and Prostaglandin E2.

### 2.3 Histology and staining

Tissues and organoids were fixed with 10% neutral‐buffered formalin (Sigma–Aldrich) at room temperature for 24 h or 0.5 h, respectively. The paraffin embedding was performed as follows: samples went through a graded‐ethanol series, xyleneand then paraffin. The embedded samples were cut into 5 μm and prepared for H&E and immunohistological (IHC) staining according to a standard protocol. For IHC, primary antibodies against CK7 (NBP2‐44814, Novus Biologicals, 1:50), histone deacetylase (HDAC1) (sc‐81598, Santa Cruz, 1:50), HDAC2 (ab32117, Abcam, 1:100), HDAC3 (ab32117, Abcam, 1:100), HDAC4 (sc‐46672, Santa Cruz, 1:50), HDAC5 (sc‐133225, Santa Cruz, 1:50), HDAC6 (ab133493, Abcam, 1:100), cleaved‐Caspase3 (CST‐9579, Cell Signaling Technology, 1:100), Ki‐67 (CST‐12202, Cell Signaling Technology, 1:100), acetyl‐Histone H3 (Lys18) (CST‐131998, Cell Signaling Technology, 1:100) and p‐AKT (CST‐4060, Cell Signaling Technology, 1:100) were used.

### 2.4 Whole‐exome sequencing analysis

Genomic DNA was isolated using DNeasy Blood & Tissue Kit (Qiagen, Germany) following the manufacturer's protocol. The SureSelect Human All Exon V6 (Agilent Technologies) was used to capture exome according to the manufacturer's guide. Qubit® 2.0 Fluoromete was used to assess the quantity of libraries. The quality and size measurement were performed by 2100 Bioanalyzer High Sensitivity DNA Assay. The paired‐end sequencing (2×150 bp) of qualified libraries were performed on Illumina HiSeq X‐ten platform. Human reference genome (hg19/GRCh37) were used to align the FASTQ files through BWA V0.7.13. The aligned files (sam/bam format) were sorted and indexed with SAMTools (version 1.3), then duplicates were flagged with Picard (version 2.2.4). Reads were locally realigned and their base qualities were recalibrated by using GATK (version 3.5). Finally, mapping statistics including depth and coverage were generated from recalibrated files by BEDTools (version 2.16.1) and in‐house perl/python scripts. Tumour mutation burden (TMB) was defined as the total somatic alterations in coding regions, and quantified as mutation numbers per megabase (Mb). Copy number variation was identified by XHMM (eXome Hidden Markov Model) v1.0.

### 2.5 RNA sequencing analysis

Total RNA isolation was performed using RNeasy mini kit (Qiagen, Germany). The TruSeq Stranded Total RNA Sample Preparation kit (Illumina, USA) was applied to construction strand‐specific libraries. In short, after enrichment and purification, the mRNA was cleaved into small pieces and then reversely transcribed into cDNA. After end repair, purification and enrichment, the cDNA library was quantified and validated with Qubit 2.0 Fluorometer (Life Technologies, USA) and Agilent 2100 Bioanalyzer (Agilent Technologies, USA). The libraries were diluted to 10 pM to generate cluster by cBot and then applied to sequencing on the Illumina HiSeq X ten. The construction and sequencing of RNA libraries were performed at Shanghai Biotechnology Corporation, China.

### 2.6 Single‐cell RNA sequencing

The libraries were sequenced on NovaSeq6000 (Illumina) using 2 × 150 chemistry. Cell Ranger 3.0.1 pipeline was used to process single‐cell RNA sequencing data following the manufacturer's instructions. By counting unique molecular identifiers (UMIs) and filtering non‐cell associated barcodes, gene–barcode matrices with the barcoded cells and gene expression counts were generated for each individual sample. The quality control and downstream analysis were performed by Seurat (v3.0.2). We excluded cells with detected genes less than 200 and features expressed in less than three cells. Then, cells with percentage of mitochondrial expression less than 20% and with detected genes less than 6000 or more than 200 were reserved for analysis. All functions were then run with default parameters, unless specified otherwise. The percentage of mitochondrial counts was used to regress out in “ScaleData” function. The “npcs” was set as 60 in “RunPCA” function. The top 22 principal components (PCs) were used for uniform manifold approximation and projection (UMAP) dimensional reduction. The same PCs and resolution at 0.6 were used to cluster cells. Cell types were defined by canonical cell markers, that is, epithelial cell adhesion molecule (EPCAM) for epithelial cells, COL1A1 and PDGFRB for mesenchymal cells, PECAM1 and CD34 for endothelial cells, CD79A and MZB1 for B cells, CD14 and CD163 for myeloid cells, TPSAB1 and KIT for Mast cells.

For sub‐clustering epithelial cells, the same procedure of normalisation, scaling, dimensionality reduction, and clustering were employed. The differential expressed genes were detected by “FindMarkers” function with logfc.threshold as 0.25, min.pct as 0.1, and Wilcoxon rank sum test. Top 50 genes of each sample and top 20 genes of each epithelial subcluster were showed by heatmap plotted with ComplexHeatmap (version 2.8.0), respectively. Gene with adjusted *p*‐value < 0.05 of normal or malignant samples were used to perform Gene Ontology enrichment with clusterProfiler(version 4.0.0). To infer the copy number variations (CNV) of epithelial cells, we used inferCNV packages with default parameters and additional sample information.

### 2.7 Drug treatment

Cells from each organoid were plated (5×10^3^/well) and compounds of indicated concentration were added into the culture medium 24 h after cell seeding. Cell viabilities were assessed by CellTiter‐Glo Luminescent assay 4 days after the treatment. Dimethyl sulfoxide (DMSO, 0.5%) were used as blank control. The relative ratio of drug treated cell viability under 0.5 was considered to be significant.

### 2.8 Assessment of staining of tissue slides

Given that HDAC inhibitors exhibited the outstanding anti‐cancer effect, among others, HDAC protein levels in human normal gallbladder and GBC tissues were evaluated. HDAC 1, 2 and 6 staining in GBC tissue was scored (C.L. L and X.W) by using the semi‐quantitative immunoreactivity scoring (IRS) system as described previously.^16^ The immunostaining intensity (category A) was as 0 (negative), 1 (weak), 2 (moderate immunostaining), and 3 (strong immunostaining). The percentage of immunoreactive cells (category B) was as 1 (< 10%), 2 (10–33%), 3 (33–66%), and 4 (> 66%). Multiplication of category A and B produced a result as IRS from 0 to 12 for each tissue. Patients with IRS 0–6 were classified to low‐expression groups, and patients with IRS 7–12 were categorized as classified to high‐expression groups.

### 2.9 Organoid‐derived tumour xenograft

All experiments involving organoid transplantations into mice were approved by the Ethical Committee and conducted following the Animal Care Facility guidelines of Second Military Medical University. GBC‐1 organoids with 2×10^6^ cells were dissociated from Matrigel, and injected subcutaneously into immunodeficient NOD.Cg‐*Prkdc^scid^ Il2rg^tm1Wjl^*/SzJ (NSG) mice (The Jackson Laboratory) at young age (*n* = 18). When the xenografts reached average volume 200 mm^3^, the mice were divided randomly into three groups of six mice in each: control (vehicle), Vorinostat and CUDC‐907 group. Drugs were dissolved in vehicle (5% DMSO and 95% corn oil), and injected intraperitoneal at a dose of 40 mg/kg every 3 days for 2 weeks. Tumour diameter was measured using a caliper, and tumour volume was calculated following: (width)^2^ × length/2. Two weeks after drug administration, the mice were sacrificed and xenografts were excised for further analysis.

### 2.10 Statistical analysis

Statistical analyses were performed with SPSS version 18.0 (SPSS Inc., Chicago, IL, USA) and R version 4.0.3 (R foundation for Statistical Computing). The association between HDAC expression and clinicopathological data was evaluated by the Chi‐square test or Fisher's exact test. Kaplan–Meier survival analysis (log‐rank test) were applied to detect the relationship between HDAC levels and overall survival (OS). We used univariate and multivariate Cox proportional hazard regression models to determine which variables affected OS. Hazard ratios (HRs) and 95% confidence intervals (CIs) were estimated. The reported *P* values were two‐sided; the statistical difference was considered significant if *P* < 0.05.

## 3 RESULTS

### 3.1 Establishment of organoids derived from human normal gallbladder, benign GBA and GBC tissues

**FIGURE 1:**
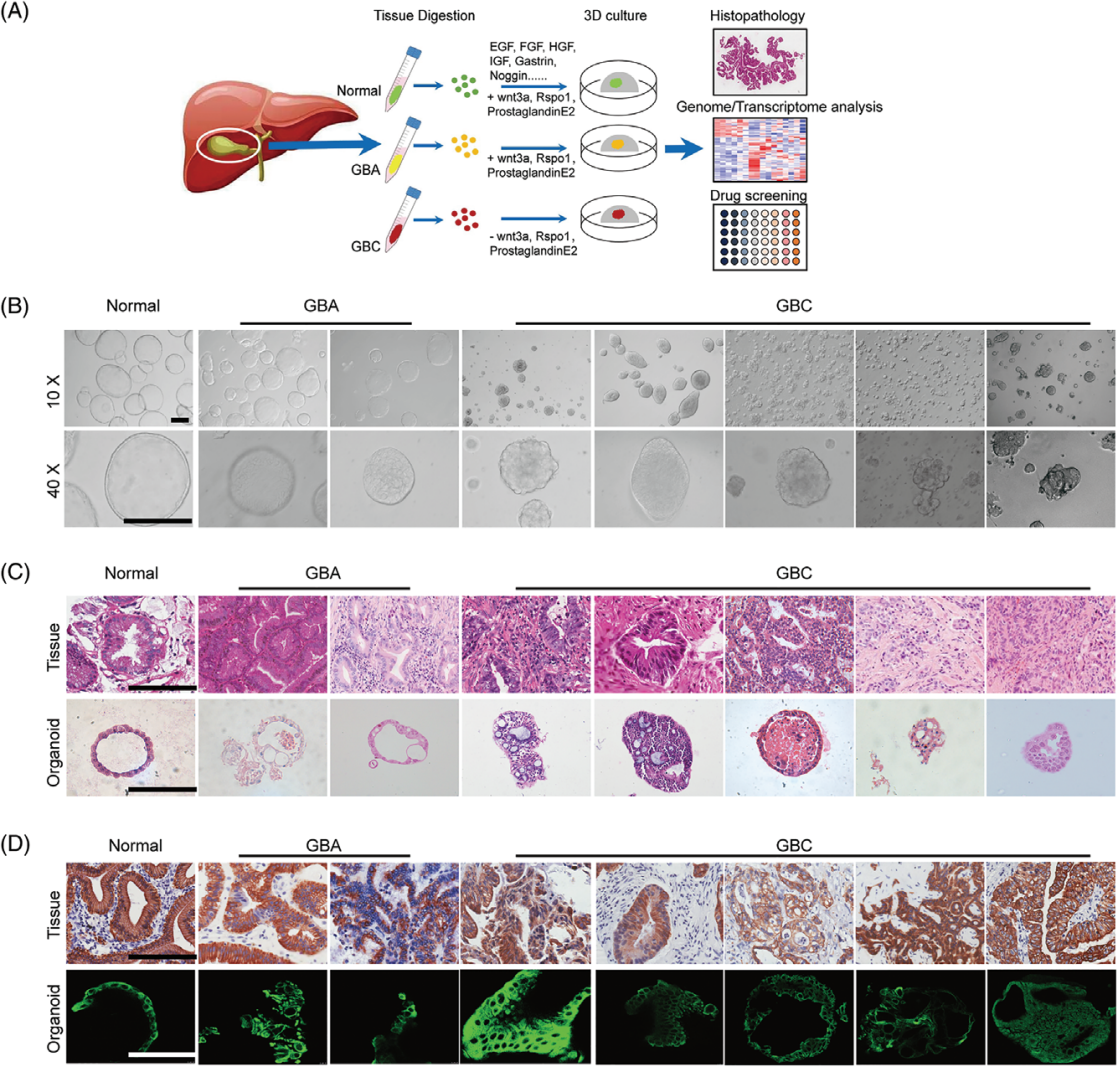
Establishment of organoids derived from human normal gallbladder, benign gallbladder adenoma (GBA) and gallbladder carcinoma (GBC) tissues (A) Experimental design. Normal gallbladder, benign GBA tissues, and GBC tissues were obtained from patients undergoing surgery (patient information detailed in Table S1) and were processed as described in the Methods. (B) Representative brightfield microscopy images of organoid lines derived from these normal gallbladder, benign GBA tissues, and GBC tissues. (C) Representative H&E staining of organoid lines and original tissues. (D) Expression of the biliary marker CK7 was detected by immunohistochemistry and immunofluorescence in the primary tissue and corresponding organoids. Scale bars: 200 μm

Due to the limited material available from surgical specimens, gallbladder tumour models are often difficult to generate. To comprehensively model the full clinical spectrum of gallbladder tumours, we collected surgically resected tumour tissues from 41 untreated GBC patients and 5 untreated GBA patients. A 3D culture protocol for gallbladder tumours was developed (Figure 1A), and five GBC organoid lines and two GBA organoids were successfully established (Table S1). These organoids were expanded for at least 10 passages and cultured stably for more than 3 months (Figure S1). We defined organoids maintained continuously for more than 3 months as successfully established. The generation efficiencies were 12.2% for GBC and 40% for GBA. Among the 36 failed cases, 19 samples failed to form organoids owing to inadequate cell number to grow into organoids; 7 cases contaminated by overgrown non‐tumourous organoids; 5 cases grew into organoids but ceased growing after several weeks; 4 cases were contaminated by bacteria or fungi; 1 organoid line failed to recover after cryopreservation. In parallel, one human normal gallbladder organoid was established from a healthy normal gallbladder, which exhibited a monolayer epithelial cystic architecture (Figure 1B). The organoids derived from GBAs and GBCs exhibited irregularly shaped cystic cribriform structures (Figure 1B). At the histological level, healthy gallbladder‐derived organoid cultures form cyst‐like hollow structures, similar to normal gallbladder tissues. In contrast, the GBA and GBC‐derived organoids displayed distinct histological and cellular architectures, with glandular domains and tumour cells growing in cribriform structures, consistent with the corresponding primary tissues (Figure 1C). IHC and immunofluorescence (IF) analysis demonstrated that all the organoids and their parental tissues retained the expression of the biliary epithelia marker CK7 (Figure 1D). These results suggest that long‐term cultured gallbladder tumouroids closely recapitulated their original tumour tissues in histological characteristic and marker expression.

### 3.2 Normal gallbladder, GBA and GBC organoids retain the genomic alterations of their original tissues

**FIGURE 2:**
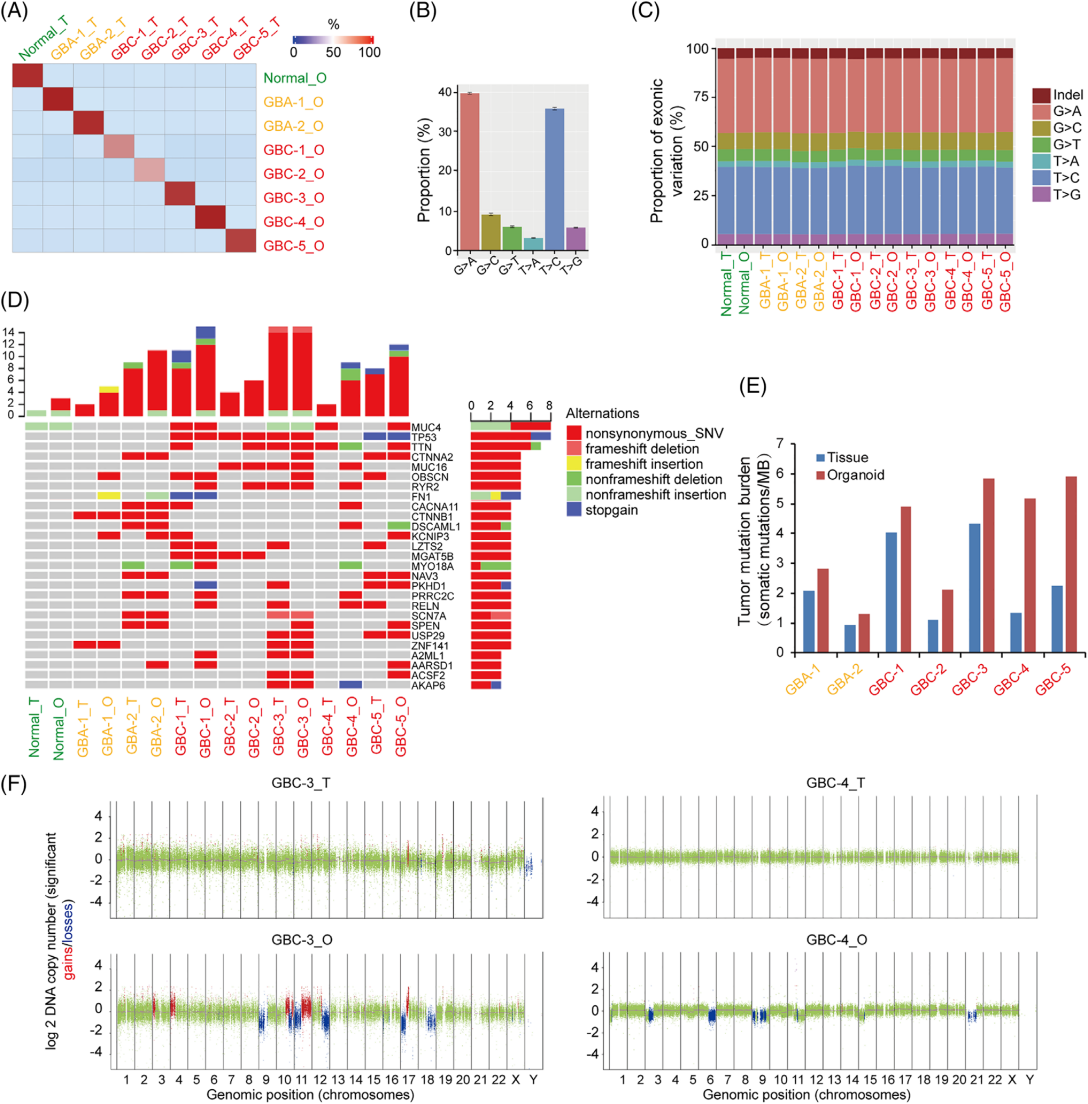
Organoids retain genetic characteristics of original tissues. (A) Correlation heat map between the variants identified in primary tissues (_T) and corresponding organoids (_O). (B) Overall distribution of base substitutions detected in all of the samples, including both organoids and the corresponding tissues. (C) Proportion of exonic variations detected in each organoid line and the corresponding tissues. (D) Overview of the mutations detected in organoid lines (O) and the corresponding tissues. (E) Tumour mutation burden (TMB) was estimated for patients with gallbladder adenoma (GBA) and gallbladder carcinoma (GBC) by counting somatic mutations, including coding single nucleotide variants (SNVs) and indels per megabase. (F) Copy number alterations (can) in representative matched primary tumour specimens and corresponding organoids. Two representative cases are shown

To determine whether organoids retain the mutational profile of their parental tissues, organoid cultures and corresponding primary tissues were subjected to whole‐exome sequencing (WES) analysis. By comparing the global genomic alterations, we found highly concordance mutational profile in organoid cultures and their original tissues, evidenced by the fact that 80%–90% of the variants in each primary tissue were retained in their paired organoid cultures (Figure 2A). The distribution of single‐base substitutions in exon for both tissues and organoids revealed overrepresentation of the G > A/C > T and T > C/A > G transversions (Figure 2B). Additionally, organoid cultures well retained the proportion of exonic variations observed in primary tissues (Figure 2C).

Among the top 27 mutated genes in organoids and the corresponding tissues (Figure 2D), we identified variations in cancer‐associated genes, including *TP53*, *MUC4*, *MUC16*, *CTNNA2*, *TTN*, *RYR2*, *MGAT5B*, *USP29* and *AKAP6* in GBC organoids and *CTNNA2*, *CTNNB1*, *KCNIP3* and *DSCAML1* in GBA organoids. Most of these gene mutations were also found in the corresponding primary tissues. Some of the mutations in GBC organoids, such as *TP53* and *MUC4*, are frequently reported genes in biliary tract cancers (BTCs).^17^, ^18^ MUC4 is an intramembrane ligand for ERBB2, one of the major drivers in BTC carcinogenesis. Our analysis shows that missense mutations of *MUC4* appeared in GBC‐1 and GBC‐4 tissues and in GBC‐1 organoids but not in normal gallbladder and GBA tissues or organoids. In addition, we identified mutations in the tumour suppressor *TP53* in 4 of 5 GBC organoids and corresponding tumour tissues. Meanwhile, several tumour‐related genes have not been reported before in BTC. For example, the *TTN* gene was suggested to be a potential oncogene in lung cancer^19^, ^20^; MGAT5B was reported to be differentially expressed or epigenetically dysregulated in a variety of cancers^21^, ^22^; and *AKAP6* mutations were demonstrated to be correlated with the susceptibility and prognosis of several cancers, including glioma, epithelial ovarian and gastric cancer.^23^, ^24^, ^25^ Notably, the results showed that GBAs already have some tumour‐related gene mutations, such as *CTNNA2, CTNNB1, and KChIP3*, which regulate the cell cycle and apoptosis. In particular, GBA‐2 contained a number of tumour‐related mutations, suggesting that some GBAs have malignant characteristics. We queried the cBioPortal database (http://www.cbioportal.org/), which provides information of large‐scale cancer genomics, and found that most of the above‐mentioned mutations present in the mutation gene list of combined GBC samples of two previous studies, for instance, *TP53* (50%), *MUC16* (18.8%), *TTN* (15.6%), *MUC4* (6.3%), *CTNNB1* (4.9%), *USP29* (3.1%), *AKAP6* (3.1%) and *DSCAML1* (3.1%).^26^, ^27^ These results suggested that organoids generated from GBAs and GBCs recapitulated the mutational landscape and tumour‐specific mutations observed in human gallbladder tumour.

To characterize the overall mutation rate in organoids and corresponding tissues, TMB was calculated. Intriguingly, GBC organoids and corresponding tissues from different patients exhibited distinctly varied TMB degrees, from 1.09 to 5.84 mutations/Mb, demonstrating high heterogeneity and low mutational rate of GBCs. Moreover, GBC organoids showed higher TMB than matched tissues, especially in GBC4 and GBC5, possibly because of non‐cancerous components and dilute tumour purity in GBC tissues (Figure 2E).^28^ Copy‐number analyses of the primary GBC tissues and corresponding organoids showed varied concordance (Figure 2F). Similar to TMB, the GBC organoids showed more copy number alterations (CNAs) than original tumour tissues. This situation have also been reported in other studies, such as breast cancer and pancreatic cancer organoids,^29^, ^30^ indicating different tumour cell purities between tissues and organoids in a variety of tumours. As most primary tumour specimens, which usually contain stromal cells such as fibroblasts, immune cells and blood vessels, have insufficient purity to reveal mutation signals, whereas organoid cultures mainly comprised of epithelium tumour cells. High purity of tumour cells in organoids may also lead to increased resolution and signal amplification. Besides, we cannot rule out the possibility that some additional mutations of environment‐sensitive genes acquired during the long‐term culture. Further investigations are needed to address this issue.

### 3.3 Transcriptomic analysis of organoid cultures and their original tissues

**FIGURE 3:**
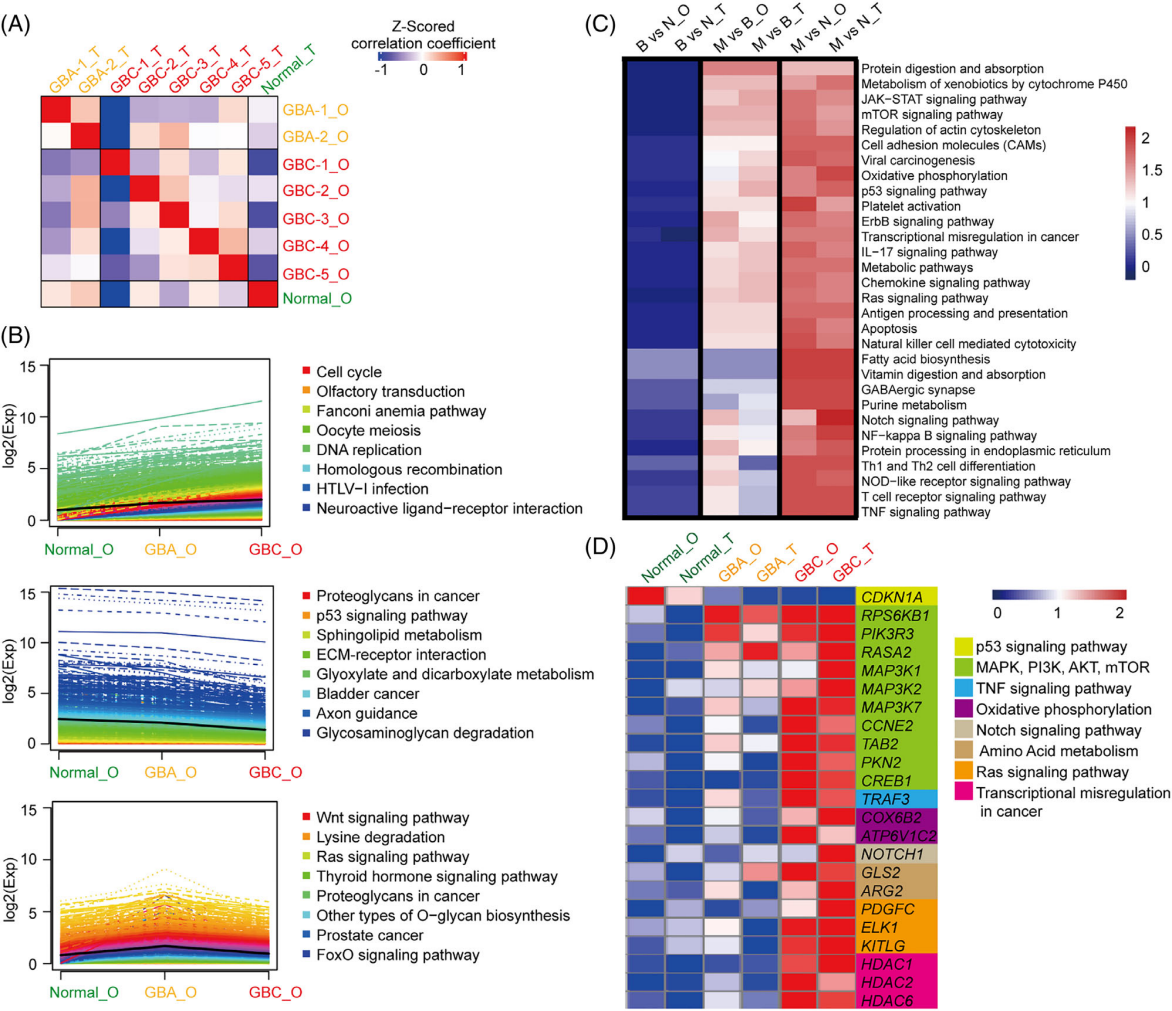
Transcriptomic analysis of organoid lines and their original tissues (A) Correlation heat map between primary tissue (_T) and paired organoid line (_O) expression profiles after at least 2 months of expansion in culture (column *z*‐scored). (B) Kyoto Encyclopaedia of Genes and Genomes (KEGG) cluster analysis of gene sets that varied across normal gallbladder, gallbladder adenoma (GBA) and gallbladder carcinoma (GBC) organoid lines. (C) Heat‐map analysis of variations between each subtype of primary tissue and organoid line (B vs. N, GBA vs. normal; M vs. B, GBC vs. GBA; M vs. N, GBC vs. normal). (D) Heat map analysis of representative genes in the indicated signaling pathways

To further characterize our organoid cultures, the gene expression profiles of organoids were compared to those of the primary tissues using RNA sequencing (RNA‐seq) analysis. Healthy gallbladder‐derived organoid and original tissue were used as controls. The correlation analysis of gene expression profiles indicated that individual organoid lines correlated to their original tissues and tumour types (Figure 3A). To compare the variations in gene expression across normal gallbladder, GBA and GBC organoids, the expression data of organoids were subjected to Kyoto Encyclopaedia of Genes and Genomes (KEGG) cluster analysis. As shown in Figure 3B, we identified a set of pathways that were gradually upregulated from normal gallbladder organoids to GBA and GBC organoids, such as cell cycle‐ and DNA replication‐related pathways. Other pathways, such as p53 signalling and extracellular matrix (ECM)‐receptor interaction pathways, were gradually downregulated from normal gallbladder organoids to GBA and GBC organoids. These gradually upregulated or downregulated pathways are closely related to the capabilities of tumour cells obtained during the tumour development, for example, unlimited replicative potential and invasion and ability for metastasis.^31^ In comparison with GBC organoids, there were also pathways that were upregulated more obviously from the normal gallbladder to GBA organoids, such as Wnt signalling, suggesting that these pathways are much more important in tumourigenesis.

Next, the differentially expressed genes (DEGs) between each group were subjected to gene set enrichment analysis (GSEA). Notably, we identified a number of signalling pathways, including the JAK‐STAT, mTOR, oxidative phosphorylation and p53 signalling pathways, that can well draw distinctions across the normal gallbladder, GBA and GBC tissues, with similar profiles between tissues and matched organoids (Figure 3C). To further confirm the expression status of specific genes in organoid lines and their corresponding parental tissues, a set of genes in signalling pathways were subjected to hierarchical clustering analysis. Consistent with previous data, abnormal and gradual activation of genes in mitogen‐activated protein kinase (MAPK) signalling (RPS6KB1, PIK3R3, MAP3K1, MAP3K2, MAP3K7 and CCNE2), PI3K‐AKT‐mTOR signalling (TAB2, PKN2, CREB1), oxidative phosphorylation pathway (COX6B2, ATP6V1C2), Ras signalling (PDGFC, ELK1 and KITLG) and HDAC1, HDAC2, HDAC6 were observed in GBA and GBC organoids, with closely resembled expression patterns of original tissues (Figure 3D). Interestingly, two or more of these cancer‐promoting pathways were coactivated in each individual GBC organoid (Figure S2). In addition, the tumour inhibitor CDKN1A (P21) was decreased in GBA and GBC, which is downstream of TP53 and acts as a determinant of cell cycle regulation (Figure 3D). These data showed that PDOs recapitulated the transcriptomic features of primary gallbladder tumours, and abnormally expressed genes might be responsible for tumour malignancy and tractable as therapeutic targets.

### 3.4 Single‐cell RNA sequencing of organoid cultures and their original tissues

**FIGURE 4:**
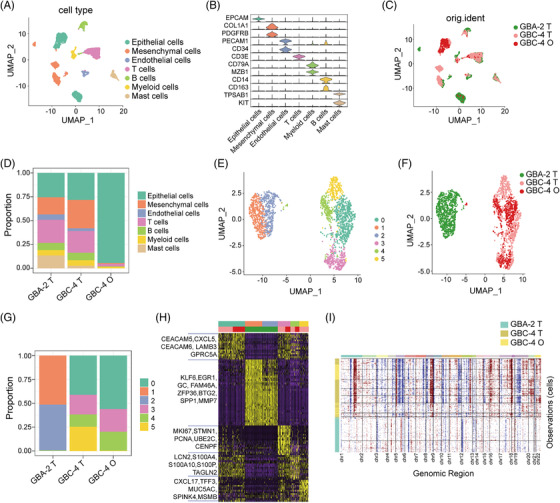
Single‐cell RNA sequencing of organoid cultures and their original tissues. (A) Uniform manifold approximation and projection (UMAP) plots showing cell types for 7124 cells. (B) Violin plots showing the smoothed expression distribution of marker genes in seven cell types. (C) UMAP plots of 7124 cells showing their origins. (D) Proportion of indicated cell‐type clusters in each sample. (E) UMAP plots showing epithelial cells, color‐coded according to different clusters. (F) UMAP plots of epithelial cells showing sample origin. (G) Proportion of subcluster cell types in each sample. (H) Heatmap of the top 20 differentially expressed genes for each epithelial cell cluster. (I) The landscape of inferred large‐scale copy number variations (CNVs) for all of the epithelial cells. The annotation tracks on the left indicate the inferred CNV clusters and corresponding sample origin. Chromosome numbers are labelled at the bottom. Red indicates copy number gain, and blue indicates copy number loss

To verify the reproducibility within the intratumour heterogeneity of organoid cultures, we generated droplet‐based single‐cell RNA sequencing (sc‐RNA seq) profiles by using three samples: GBA‐2 tissue (GBA‐2_T), GBC‐4 tissue (GBC‐4_T) and matched GBC‐4 organoid (GBC‐4_O). After quality control and doublet removal, we obtained a total of 7124 single cells. Following gene expression normalisation, we employed the UMAP method to reduce the dimensionality. These cells were assigned to seven major distinct cell‐type clusters (Figure 4A), which were annotated by canonical marker genes: epithelial cells (EPCAM^+^); mesenchymal cells (COL1A1^+^ and PDGFRB^+^); endothelial cells (PECAM1^+^ and CD34^+^); T cells (CD3E^+^); B cells (CD79A^+^ and MZB1^+^); myeloid cells (CD14^+^ and CD163^+^); and mast cells (TPSAB1^+^ and KIT^+^) (Figure 4B). GBA‐2_T and GBC‐4_T consisted of distinct cell‐type clusters, while matched GBC‐4_O was composed predominately of epithelial cells (Figure 4C,D). We further examined the heterogeneity of epithelial cell populations within and across samples. In total, epithelial cells comprised six major subsets, and remarkably, these epithelial cells reclustered by their patient of origin (Figure 4E). Subclusters 1 and 2 were almost entirely comprised of epithelial cells from the benign GBA‐2 tissue. Subclusters 0, 3, 4 and 5 were tumour specific and evenly shared by cancerous GBC‐4 tissue and its paired organoid (Figure 4F,G). Analyses of the DEGs showed that epithelial cells from benign and malignant lesions had distinct expression patterns, while a high degree of consistency was observed between GBC‐4_T and matched GBC‐4_O (Figure S3). We further conducted DEG analyses to characterize the six epithelial subsets (Figure 4H). Labelled by benign signatures, subclusters 1 and 2 similarly overexpressed metabolism‐related genes and tumour suppressors (KLF6, EGR1, GC, FAM46A, ZFP36, BTG2, etc.). Of note, GBC‐4 tissue and its corresponding organoid shared similar DEGs across subclusters 0, 3 and 4. Subcluster 0 overexpressed adhesion‐ and migration‐related genes (CEACAM5, CEACAM6, CXCL5, GPRC5A, LAMB3, etc.). Subcluster 3 overexpressed proliferation‐related genes (MKI67, STMN1, PCNA, UBE2C and CENPF). Subcluster 4 overexpressed epithelial–mesenchymal transition (EMT)‐related genes (LCN2, S100A4, S100A10, S100P and TAGLN2). Subcluster 5, distinctly derived from the tumour tissue, overexpressed mucus secretion‐related genes (CXCL17, TFF3, MUC5AC, SPINK4 and MSMB, etc.). By inferring large‐scale chromosomal CNVs, a diverse spectrum of chromosomal aberrations was present across epithelial cells and those from benign GBA‐2 with much lower inferred CNVs (Figure 4I). However, the inferred CNVs were highly consistent between epithelial cells from GBC‐4 tissue and matched organoids. Together, these data indicated that GBC‐derived organoids could recapitulate the transcriptional features and heterogeneity of their derived tissue at single‐cell resolution.

### 3.5 GBC organoids for in vitro patient‐specific drug trials

**FIGURE 5:**
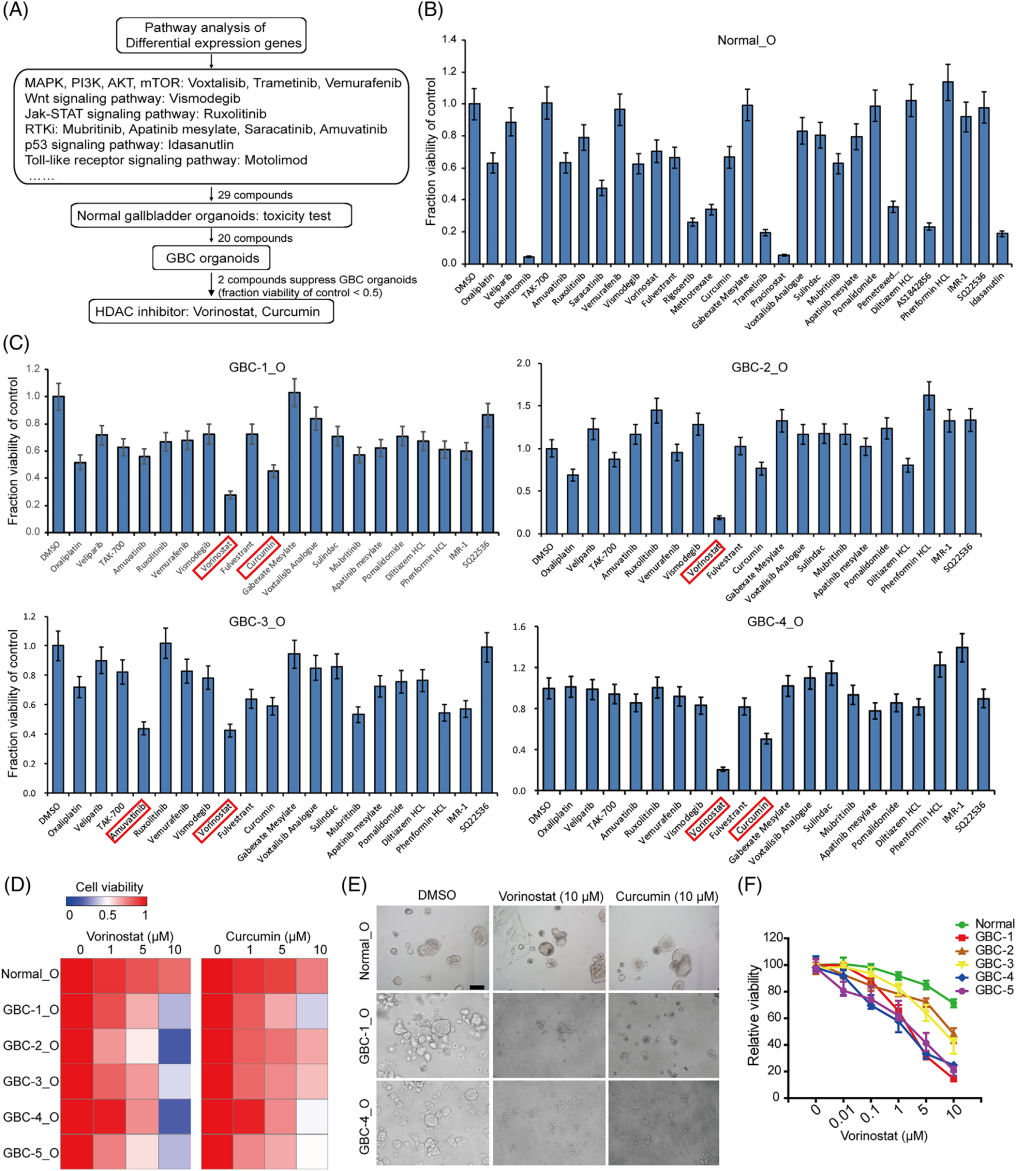
Gallbladder carcinoma (GBC) organoids for in vitro drug screening. (A) Drug screening design. From a library of 29 compounds that targeted the most active signalling pathways in GBC, we identified two compounds that were able to significantly suppress GBC organoids. (B) Effects of 29 compounds on the growth of normal gallbladder organoids. (C) Effects of 20 compounds on the growth of GBC organoids, which had no or low toxicity on normal gallbladder organoids. (D) Heatmap of the viability of GBC organoids after 4 days of treatment with vorinostat and curcumin. (E) Representative brightfield microscopy images of normal and GBC organoids after 4 days of treatment with vorinostat and curcumin at 10 μM. Scale bars: 200 μm. (F) Cell viability of normal and GBC organoids after 4 days’ treatment with vorinostat at different concentrations

To develop more effective drugs against GBCs, we selected 29 compounds (approved for clinical use by the food‐and‐drug‐administration (FDA)) targeting signalling pathways commonly activated in tumours and assessed their viability to suppress the viability of GBC organoids, for example, MAPK, Janus Kinase‐signal transducer and activator of transcription (JAK‐STAT), receptor tyrosine kinase and HDAC inhibitors (Figure 5A, Table S2). The toxicity of these inhibitors on normal gallbladder organoids was first investigated. As shown in Figure 5B, nine compounds (e.g., delanzomib, trametinib and pracinostat) showed significant suppressive effects against normal organoids, which were excluded from the following test. We further applied the remaining 20 compounds to examine the drug response of GBC organoids. It should be noted that different GBC organoids exhibit varied drug responses, and all of these GBC organoids were broadly resistant to various inhibitors targeting JAK, PI3K, MAPK, RAF, PARP, AMPK and Hedgehog. Amuvatinib, a multitargeted kinase inhibitor against c‐KIT, PDGFRα and Flt3, only exhibited a suppressive effect in GBC‐3 organoids (Figure 5C,D), and the GBC organoids showed a loss of 3D structure and viability after 4 days of treatment at a dose of 10 μM (Figure 5E). However, vorinostat and curcumin, HDAC inhibitors, effectively restrained the growth of GBC organoids (Figure 5C). Furthermore, various doses of vorinostat treatment in organoid lines demonstrated the suppressive effects on GBC organoids in a dose‐dependent manner, with IC50 (half maximal inhibitory concentration) from 1.11 to 18.05 μM (Figure 5F), suggesting that HDAC inhibitors are potential drugs against GBC organoids.

### 3.6 Overexpression of HDAC1, ‐2, and ‐6 in GBC tissues predicts a poor prognosis

**FIGURE 6:**
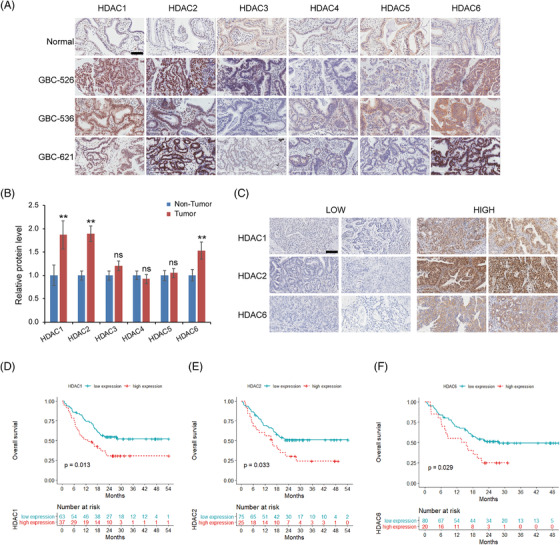
Upregulation of histone deacetylase (HDAC1), ‐2, and ‐6 in gallbladder carcinoma (GBC) is correlated with a poor prognosis. (A) Expression of HDACs in normal gallbladder and GBC tissues was tested by immunohistochemical staining. Representative IHC staining of HDACs in there GBC tissues and one normal gallbladder. Scale bars: 100 μm. (B) The HDACs expression in 10 normal gallbladder and 100 GBC specimens was quantified with ImageJ software. Data are presented as the mean ± SD (** *P* < 0.01). (C) Representative micrographs showing low‐level and high‐level expression of HDAC1, HDAC2 and HDAC6 in GBC tissues. Scale bars: 100 μm. (D–F) Survival plots for groups with high and low expression of HDAC1 (D), HDAC2 (E) and HDAC6 (F) in GBC specimens

HDACs have been reported to play significant roles in human malignant tumour development and progression,^32^, ^33^ and HDAC inhibitors are currently being investigated as anticancer agents in clinical trials. The single‐cell regulatory network inference and clustering (SCENIC) analysis identified HDAC2 as one of the underlying transcription factors in epithelial cells from malignant GBC‐4 tissue and matched organoid (Figure S4). To explore the clinical significance of HDACs expression in GBC, we performed IHC staining in human GBC (100 samples) and normal gallbladder (10 samples) tissues. The expression levels of HDAC1, ‐2 and ‐6 were obviously upregulated in GBC tissues in comparison with normal gallbladder tissues (Figure 6A,B). Based on the IHC results of HDAC1, ‐2 and ‐6 expression in tumour tissues, all 100 GBC samples were categorized into low‐expression and high‐expression groups (Figure 6C). Statistical analysis showed that the high‐HDAC1 expression group had worse nodal status (*P* = 0.003) and advanced tumor node metastasis (TNM) stage (*P* = 0.01); the high‐HDAC2 expression group had more patients with liver invasion (*P* = 0.03, Table S3). Kaplan–Meier analyses indicated that patients with high HDAC 1, 2 and 6 levels had substantially shorter OS (*P* = 0.013, 0.033 and 0.029, respectively; Figure 6D–F). Multivariate analysis further revealed that HDAC1 overexpression was an independent prognostic predictor (Table S4). For other clinicopathological features, only distant metastasis (M1) and poor differentiation had significant prognostic influences. These findings demonstrated HDAC1, ‐2 and ‐6 as predictors for a poor prognosis of GBC, which further indicated the therapeutic value of HDAC inhibitors.

### 3.7 Coinhibition of HDAC and other tumour‐promoting signalling pathways synergically suppresses GBC organoid growth

**FIGURE 7:**
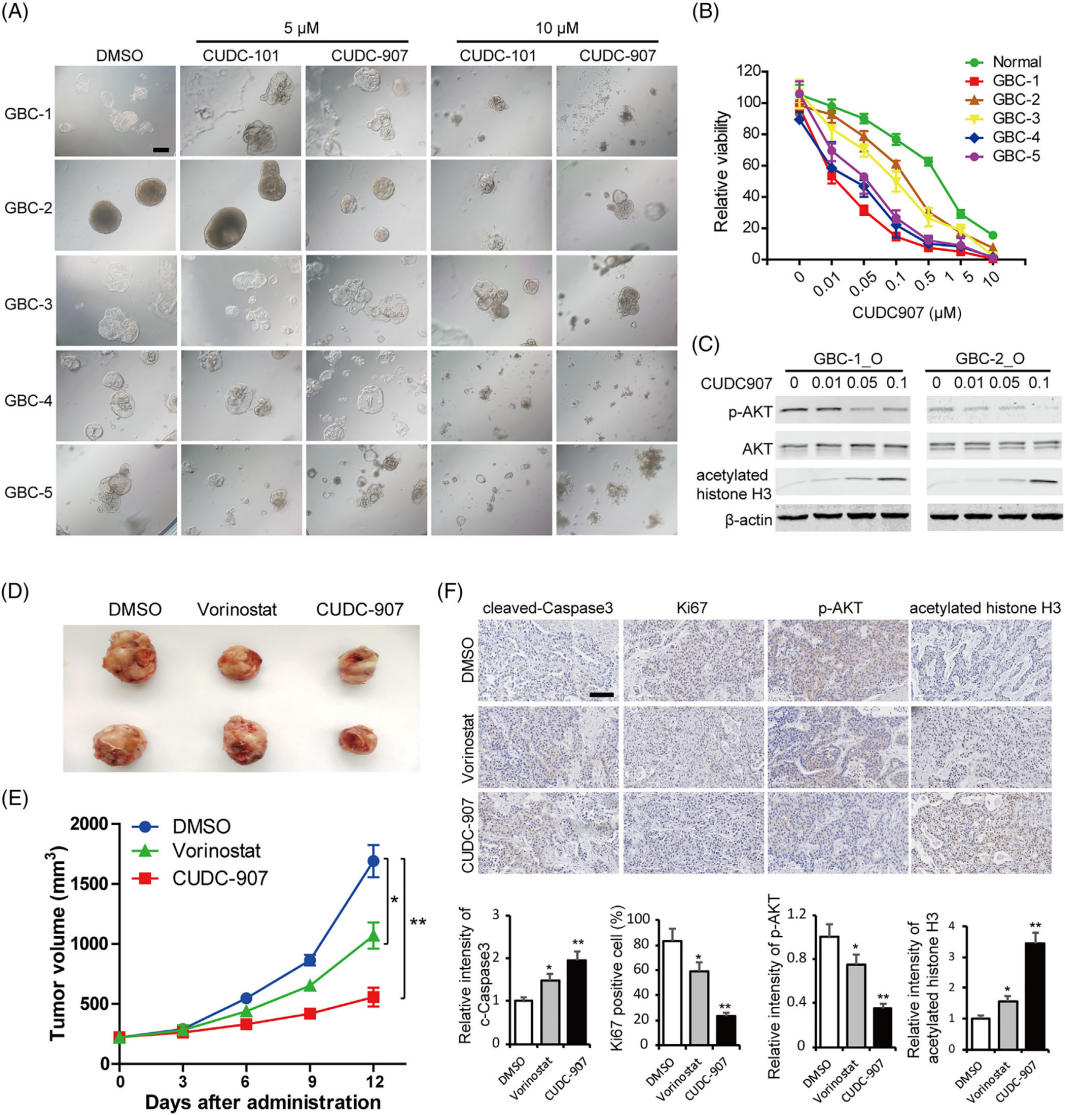
Dual histone deacetylase (HDAC) and tumour‐promoting pathway inhibitors suppress gallbladder carcinoma (GBC) organoid growth. (A) Representative brightfield microscopy images of GBC organoids after 4 days of treatment with CUDC101 and CUDC907 at 5 μM and 10 μM, respectively. Scale bars: 200 μm. (B) Cell viability of normal and GBC organoids after 4 days’ treatment with CUDC907 at different concentrations. (C) The expression levels of phosphor‐AKT, AKT and acetylated histone H3 were determined by western blot, and β‐actin was used as a loading control. (D) The GBC organoids were inoculated subcutaneously into nude mice. Representative images of the xenograft tumours. (E) The tumour volumes were measured, and the results are expressed as the mean ± standard deviation (SD). * *P* < 0.05, ** *P* < 0.01. (F) Immunohistological (IHC) staining of cleaved Caspase 9, Ki67, phospho‐AKT and acetylated histone H3 in the tumour tissues from each group. Scale bars: 100 μm

In view of the common coactivation of HDACs and other cancer‐promoting pathways in GBC, such as PI3K‐AKT and Ras signaling, we aimed to determine whether combined inhibition of these pathways with HDACs has synergistic antitumour effects against GBC patients. The novel dual epidermal growth factor receptor (EGFR) and HDAC inhibitor CUDC‐901 and dual PI3K and HDAC inhibitor CUDC‐907 were employed to treat GBC organoids. Both CUDC‐101 and CUDC‐907 exhibited remarkable inhibition of organoid growth in vitro (Figure 7A). According to cell survival curves, the IC50 of CUDC‐907 on GBC organoids is from 12.76 to 116.9 nM (Figure 7B), indicating a specific activity for GBC growth‐inhibition. Consistently, the protein expression of phosphorylated AKT were downregulated and acetylated histone H3 (substrate of HDACs) were increased after CUDC‐907 administration in GBC organoids (Figure 7C).

Moreover, treatment with vorinostat or CUDC‐907 consistently resulted in tumour growth inhibition in GBC organoid‐derived xenograft models, and as expected, the PI3K‐HDAC dual inhibitor CUDC‐907 displayed stronger antitumour activity than vorinostat (Figure 7D,E). In addition to decreased tumour size, CUDC‐907 administration induced higher expressions of cleaved Caspase 3 and lower expressions of Ki67, indicating promoted cell apoptosis and suppressed cell proliferation (Figure 7F). Meanwhile, IHC staining indicated that CUDC‐907 treatment significantly restrained AKT and HDAC activities evidenced by reduced phosphor‐AKT and increased histone H3 acetylation (Figure 7F). These results demonstrated that coinhibition of cancer‐promoting pathways and HDACs may be a viable therapeutic strategy for GBC patients.

## 4 DISCUSSION

GBC is a malignancy arising on the basis of chronic gallbladder diseases and is often diagnosed late with a poor prognosis. A better understanding of GBC progression could provide potential therapies and prolong survival. Studies on GBC have thus far been restricted due to a lack of in vitro models. Here, we described a method for the successful generation of organoids that generally recapitulates the characteristics of original normal gallbladder, benign GBA and malignant GBC tissues.

Recently, several groups have generated organoids from normal gallbladder and gallbladder cancer specimens.^34^, ^35^, ^36^ Saito et al. tried to establish organoids using five GBC specimens. However, only one GBC organoid was established.^35^ To date, organoids derived from benign GBA have not been reported. Additionally, our present study analysed the differences in culture condition, morphology, genetic mutation and gene expression across normal gallbladder, benign GBA and malignant GBC organoids, which will help us understand the malignant progression of human GBC.

Insulin‐like growth factor (IGF) is a peptide hormone critical for growth, development and oncogenic transformation.^37^ IGF signalling plays important roles in cancer formation, progression and metastasis in a number of malignant tumours, including gallbladder cancer.^38^, ^39^ Recently, Fujii et al. reported that IGF‐1 together with other growth factors, such as EGF, HGF or FGF, profoundly promotes organoid growth.^40^ In the present study, to increase the success rate for the establishment of our organoids, we supplemented IGF on the basis of previously reported mediums for human liver organoids and nontumoural gallbladder organoids. Moreover, proteins linked to Wnt signalling, including Wnt3a, R‐spondin and prostaglandin E2, were added to the medium of normal gallbladder and benign GBA organoids but not to the medium of GBC organoids. Prostaglandin E2 has been reported to prevent detachment‐induced cell death and activate the canonical Wnt signalling cascade,^41^, ^42^ and prostaglandin E2 has been used to support the growth of colonic and intestinal organoids.^43^, ^44^

By using the modified mediums, success rates for the establishment of normal gallbladder, benign GBA and malignant GBC organoids were 50% (1/2), 40% (2/5) and 12.2% (5/41), respectively. In fact, the success rate for GBC organoids establishment in our study is far from satisfactory. As most GBCs originate from chronic inflammation, there are large amounts of fibrous connective tissue in tumour tissues. This usually makes the tumour tissues very difficult to digest. After digestion, only a small number of epithelial cells were obtained and the cell vitality is quite low. The contamination of non‐tumourous organoids is also an important reason for the low generation rate. In future, the generation protocol and culture conditions need to be optimized to increase generation efficiency, for example, the approach reported by Tuveson's group for generation of pancreatic cancer organoids.^45^

In our study, the similarities in morphology and genetic and transcriptional characteristics between organoids and parental tissues demonstrated that established organoids faithfully recapitulate the histological complexity and molecular features of human tissues. More importantly, GBC organoid cultures maintained intratumoural cellular heterogeneity of their derived tissue at the single‐cell level, thus representing an ideal preclinical model for pathological mechanism research and personalized drug screening.

In the present study, we identified multiple gene mutations in gallbladder tumour organoids, some of which have been reported in biliary tract tumours (e.g., *TP53* and *MUC4*), while the others have not (e.g., *TTN*, *MGAT5B* and *AKAP6*). It is of interest to note that GBA organoids already have mutations in some cancer‐related genes (e.g., *CTNNA2* and *CTNNB1*), suggesting the malignancy potential of some GBA cells. By comparing the gene expression profiles of organoids, we showed that a series of signaling pathways varied across normal gallbladder, GBA and GBC organoids, for example, JAK‐STAT, PI3K‐AKT, oxidative phosphorylation and TP53 pathways, which are implicated in cell proliferation ability and tumoural malignancy. The abnormal expression profiling of genes associated with these pathways in GBA and GBC organoids was also identified, such as MAP3K1, MAP3K2 (MAPK signaling), TAB2, PKN2 (PI3K‐AKT signaling), COX6B2, ATP6V1C2 (oxidative phosphorylation pathway), and HDACs. Although the exact role of these genes and pathways in the progression from chronic gallbladder disease to malignant carcinomas needs further exploration, our findings provide information on the genotypic and phenotypic changes at the organoid level, which may helpful in understanding the complex mechanisms and defining drug targets of gallbladder tumours.

Most GBC patients are diagnosed at a late, unresectable stage; therefore, systemic chemotherapy is an important therapeutic strategy for advanced GBC patients. Gemcitabine, fluoropyrimidines and platinum compounds are the most frequently used chemotherapeutics to treat GBC, similar to chemotherapy options for cholangiocarcinoma.^46^ However, these nontargeted anticancer agents usually have limited effectiveness, with an OS time of < 1 year, and cause severe adverse effects.^47^ While clinical trials for targeted therapeutics in GBCs using EGFR, Vascular Endothelial Growth Factor Receptor (VEGFR), MEK and PI3K inhibitors have been completed, these randomized trials failed to disclose the superiority of any targeted drugs with chemotherapy.^48^, ^49^, ^50^ In line with the clinical results, our established GBC organoids were largely heterogeneous and highly resistant to multiple targeted drugs approved by the FDA for tumour therapy, such as JAK‐STAT, protein kinase B, PKB (PI3K‐AKT) and PARP inhibitors. Interestingly, gene expression profiling analysis revealed that the MAPK, PI3K‐AKT, and Ras pathways and HDACs were always activated simultaneously in GBC organoids, which may provide an explanation for the resistance to agents targeting one molecule or pathway.

In view of HDACs being implicated in a variety of cancers, including cholangiocarcinoma and GBC, different kinds of HDAC inhibitors are being investigated in preclinical or clinical studies as anticancer agents. Promising antitumour activity has been noted with HDAC inhibitors in several human tumours, such as hepatocellular carcinoma, breast cancer and head and neck squamous cell carcinoma.^51^, ^52^ Here, our study showed upregulated expression and prognostic value of HDAC1, ‐2 and ‐6 in GBC tissues. Consistently, HDAC inhibitors (vorinostat and curcumin), especially dual HDAC and cancer‐promoting pathway inhibitors (CUDC‐901 and CUDC‐907), exhibited growth‐inhibitory effects across different GBC organoids with low toxicity to normal gallbladder organoids, suggesting a safe and effective treatment regimen in GBC. Further studies, especially random clinical trials, will be needed to examine the potential clinical application of these drugs for GBC therapy.

In summary, we herein established organoids that generally recapitulate the morphology, genetic and transcriptional characteristics, and intratumour heterogeneity of normal gallbladder, benign GBA and malignant GBC tissues. Moreover, our study provides important insights into the genetic and molecular changes linked to carcinogenesis and the progression of gallbladder tumours. However, there are several aspects that are needed to be improved in future studies to better employ organoids technology in GBC research. Most importantly, more normal specimens and larger GBC sample sizes are warranted. In addition, establish protocols and culture conditions of organoids are required to be optimized to increase efficiencies. The histological, transcript and genomic changes of organoid during long‐term culture should be better monitored.

## Supporting information

Supporting Information
