# Supplementary material for: Patient‐derived organoids for personalized gallbladder cancer modelling and drug screening
Source: Clin Transl Med. 2022 Jan 24;12(1):e678. doi: 10.1002/ctm2.678 (PMC8786696; doi:10.1002/ctm2.678)

**Supplementary Table1. The list of clinical samples used in research.**

| **Patient sample** | **Age (years)** | **Gender** | **HBsAg** | **Gallstone** | **Pathological Diagnosis** | **Differentiation** | **Metastasis** | **Tumor size (cm)** | **TNM stage** |
| --- | --- | --- | --- | --- | --- | --- | --- | --- | --- |
| **Normal** | 53 | male | Negative | yes | cholecystitis | / | / | / | / |
| **GBA-1** | 69 | male | Positive | yes | adenoma | / | / | uncertain | / |
| **GBA-2** | 49 | female | Negative | yes | adenoma | / | / | uncertain | / |
| **GBC-1** | 61 | female | Negative | yes | adenocarcinoma | moderate | no | 6 | T2bN0M0 IIA |
| **GBC-2** | 55 | female | Negative | yes | adenocarcinoma | moderate | liver metastasis | 2 | T3N2M1 IVB |
| **GBC-3** | 49 | male | Negative | no | adenocarcinoma | moderate | no | uncertain | T3N2M0 IVB |
| **GBC-4** | 64 | female | Negative | no | adenocarcinoma | moderate | no | 5 | T3N1M1 IVB |
| **GBC-5** | 76 | male | Negative | yes | adenocarcinoma | poor | liver metastasis | 4 | T3N0M0 IIIA |

**Supplementary Table 2. Inhibitors used in organoids drug screening**

| **No.** | **Inhibitor** | **Pathway** | **Target** |  | |  |
| --- | --- | --- | --- | --- | --- | --- |
| 1 | Oxaliplatin | DNA synthesis | DNA synthesis | |  |  |
| 2 | Rigosertib (ON-01910) | Cell cycle | PLK |  | |  |
| 3 | Pemetrexed Disodium Hydrate | Proximal tubule bicarbonate reclamation | DHFR |  | |  |
| 4 | Voxtalisib Analogue | PI3K-Akt signaling pathway | mTOR,PI3K | |  |  |
| 5 | Trametinib (GSK1120212) | MAPK signaling pathway | MEK |  | |  |
| 6 | Vemurafenib | MAPK signaling pathway | Raf |  | |  |
| 7 | Vismodegib (GDC-0449) | Wnt signaling pathway | Hedgehog/Smoothened | |  |  |
| 8 | Ruxolitinib (INCB018424) | Jak-STAT signaling pathway | JAK |  | |  |
| 9 | Mubritinib (TAK 165) | ErbB signaling pathway | HER2 |  | |  |
| 10 | Apatinib mesylate | VEGF signaling pathway | VEGFR |  | |  |
| 11 | Idasanutlin (RG-7388) | p53 signaling pathway | Mdm2 |  | |  |
| 12 | Veliparib (ABT-888) | Apoptosis | PARP |  | |  |
| 13 | Saracatinib (AZD0530) | Tight junction | Src |  | |  |
| 14 | Amuvatinib (MP-470) | Pathways in cancer | c-Kit,FLT3,PDGFR | |  |  |
| 15 | IMR-1 | Notch signaling pathway | Notch | |  |  |
| 16 | Delanzomib (CEP-18770) | Proteasome | Proteasome | |  |  |
| 17 | Sulindac | Lysosome | COX |  | |  |
| 18 | Pomalidomide | TNF signaling pathway | TNF-alpha | |  | |
| 19 | Pracinostat (SB939) | Alcoholism | HDAC |  | |  |
| 20 | Curcumin | NF-kappa B signaling pathway | HDAC, NF-κB | |  |  |
| 21 | Vorinostat (SAHA, MK0683) | Regulation of autophagy | HDAC | |  |  |
| 22 | Fulvestrant | Estrogen signaling pathway | Estrogen/progestogen Receptor | |  |  |
| 23 | Phenformin HCL | AMPK signaling pathway | AMPK | |  |  |
| 24 | AS1842856 | FoxO signaling pathway | Foxo1 | |  |  |
| 25 | TAK-700 (Orteronel) | Metabolism of xenobiotics by cytochrome P450 | P450 (e.g. CYP17) | |  |  |
| 26 | Methotrexate | Folate biosynthesis | DHFR |  | |  |
| 27 | Diltiazem HCL | Calcium signaling pathway | Calcium channel | |  |  |
| 28 | Gabexate Mesylate | Glycine, serine and threonine metabolism | Serine Protease | |  |  |
| 29 | SQ22536 | cAMP signaling | Adenylyl cyclase | |  |  |

**Supplementary Table 3.** **Clinicopathological characteristics of 100 gallbladder cancer patients**

|  | Total | HDAC1 expression |  |  | HDAC2 expression |  |  | HDAC6 expression |  |  |
| --- | --- | --- | --- | --- | --- | --- | --- | --- | --- | --- |
|  |  | Low | High | P | Low | High | P | Low | High | P |
| **All cases** | 100 | 63 | 37 |  | 75 | 25 |  | 80 | 20 |  |
| **Gender** |  |  |  | 0.81 |  |  | 0.19 |  |  | 0.68 |
| Female | 61 | 39 | 22 |  | 43 | 18 |  | 48 | 13 |  |
| Male | 39 | 24 | 15 |  | 32 | 7 |  | 32 | 7 |  |
| **Age (years)** |  |  |  | 0.32 |  |  | 0.73 |  |  | 0.84 |
| < 65 | 53 | 31 | 22 |  | 39 | 14 |  | 42 | 11 |  |
| ≥65 | 47 | 32 | 15 |  | 36 | 11 |  | 38 | 9 |  |
| **Anti-HBc status** |  |  |  | 0.23 |  |  | 0.05 |  |  | 0.54 |
| Negative | 41 | 23 | 18 |  | 35 | 6 |  | 34 | 7 |  |
| Positive | 59 | 40 | 19 |  | 40 | 19 |  | 46 | 13 |  |
| **Gallstone** |  |  |  | 0.82 |  |  | 0.48 |  |  | 0.42 |
| No | 58 | 36 | 22 |  | 42 | 16 |  | 48 | 10 |  |
| Yes | 42 | 27 | 15 |  | 33 | 9 |  | 32 | 10 |  |
| **Gallbladder polyps** |  |  |  | 0.11* |  |  | 0.36* |  |  | 1.00* |
| No | 89 | 59 | 30 |  | 65 | 24 |  | 71 | 18 |  |
| Yes | 11 | 4 | 7 |  | 10 | 1 |  | 9 | 2 |  |
| **CEA level** |  |  |  | 0.34 |  |  | 0.80 |  |  | 0.28 |
| ≤5 ng/ml | 70 | 42 | 28 |  | 53 | 17 |  | 54 | 16 |  |
| > 5 ng/ml | 30 | 21 | 9 |  | 22 | 8 |  | 26 | 4 |  |
| **CA 19-9 level** |  |  |  | 0.65 |  |  | 0.29 |  |  | 0.84 |
| ≤37 U/ml | 43 | 26 | 17 |  | 30 | 13 |  | 34 | 9 |  |
| > 37 U/ml | 57 | 37 | 20 |  | 45 | 12 |  | 46 | 11 |  |
| **Location** |  |  |  | 0.87 |  |  | 0.31† |  |  | 0.77† |
| Fundus | 26 | 17 | 9 |  | 19 | 7 |  | 22 | 4 |  |
| Neck | 46 | 28 | 18 |  | 38 | 8 |  | 37 | 9 |  |
| Body | 17 | 10 | 7 |  | 11 | 6 |  | 13 | 4 |  |
| Diffused | 11 | 8 | 3 |  | 7 | 4 |  | 8 | 3 |  |
| **Type of surgery** |  |  |  | 0.30† |  |  | 0.08† |  |  | 0.29† |
| Standard | 45 | 29 | 16 |  | 32 | 13 |  | 37 | 8 |  |
| Combined BD resection | 26 | 18 | 8 |  | 23 | 3 |  | 23 | 3 |  |
| Extended resection | 10 | 7 | 3 |  | 7 | 3 |  | 7 | 3 |  |
| Palliative surgery | 17 | 7 | 10 |  | 13 | 4 |  | 12 | 5 |  |
| Incidental GBC redo-surgery | 2 | 2 | 0 |  | 0 | 2 |  | 1 | 1 |  |
| **Margin** |  |  |  | 0.59 |  |  | 0.59 |  |  | 0.68* |
| R0 | 76 | 49 | 27 |  | 58 | 18 |  | 62 | 14 |  |
| R1/R2 | 24 | 14 | 10 |  | 17 | 7 |  | 18 | 6 |  |
| **Liver invasion** |  |  |  | 0.87 |  |  | **0.03** |  |  | 0.84 |
| No | 47 | 30 | 17 |  | 40 | 7 |  | 38 | 9 |  |
| Yes | 53 | 33 | 20 |  | 35 | 18 |  | 42 | 11 |  |
| **Vascular invasion** |  |  |  | 0.37 |  |  | 0.42* |  |  | 1.00* |
| No | 85 | 52 | 33 |  | 62 | 23 |  | 68 | 17 |  |
| Yes | 15 | 11 | 4 |  | 13 | 2 |  | 12 | 3 |  |
| **Bile duct invasion** |  |  |  | 0.97 |  |  | 0.20 |  |  | 0.42 |
| No | 57 | 36 | 21 |  | 40 | 17 |  | 44 | 13 |  |
| Yes | 43 | 27 | 16 |  | 35 | 8 |  | 36 | 7 |  |
| **Tumor stage** |  |  |  | 0.22† |  |  | 0.45† |  |  | 0.95† |
| T0 | 2 | 0 | 2 |  | 1 | 1 |  | 2 | 0 |  |
| T1 | 9 | 5 | 4 |  | 8 | 1 |  | 8 | 1 |  |
| T2 | 0 | 0 | 0 |  | 0 | 0 |  | 0 | 0 |  |
| T3 | 68 | 46 | 22 |  | 49 | 19 |  | 53 | 15 |  |
| T4 | 21 | 12 | 9 |  | 17 | 4 |  | 17 | 4 |  |
| **Nodal status** |  |  |  | **0.003** |  |  | 0.86 |  |  | 0.75 |
| N0 | 41 | 23 | 18 |  | 32 | 9 |  | 34 | 7 |  |
| N1 | 33 | 28 | 5 |  | 25 | 8 |  | 25 | 8 |  |
| N2 | 25 | 11 | 14 |  | 18 | 7 |  | 20 | 5 |  |
| **Stage of metastasis** |  |  |  | 0.78* |  |  | 1.00* |  |  | 0.81* |
| M0 | 89 | 57 | 32 |  | 67 | 22 |  | 72 | 17 |  |
| M1 | 11 | 6 | 5 |  | 8 | 3 |  | 8 | 3 |  |
| **TNM stage** |  |  |  | **0.01**† |  |  | 0.28† |  |  | 1.00† |
| Stage 0 | 2 | 0 | 2 |  | 1 | 1 |  | 2 | 0 |  |
| Stage I | 8 | 4 | 4 |  | 8 | 0 |  | 7 | 1 |  |
| Stage II | 0 | 0 | 0 |  | 0 | 0 |  | 0 | 0 |  |
| Stage IIIA | 28 | 18 | 10 |  | 21 | 7 |  | 22 | 6 |  |
| Stage IIIB | 23 | 20 | 3 |  | 16 | 7 |  | 18 | 5 |  |
| Stage IVA | 11 | 8 | 3 |  | 10 | 1 |  | 9 | 2 |  |
| Stage IVB | 28 | 13 | 15 |  | 19 | 9 |  | 22 | 6 |  |
| **Differentiation** |  |  |  | 0.85 |  |  | 0.77 |  |  | 0.06† |
| Poorly differentiated | 24 | 16 | 8 |  | 18 | 6 |  | 16 | 8 |  |
| Moderately differentiated | 64 | 39 | 25 |  | 49 | 15 |  | 52 | 12 |  |
| Well differentiated | 12 | 8 | 4 |  | 8 | 4 |  | 12 | 0 |  |
| **Histology** |  |  |  | 0.16† |  |  | 0.44† |  | 0.19† |  |
| Adenocarcinoma | 86 | 57 | 29 |  | 65 | 21 |  | 69 | 17 |  |
| Mixed tumor type | 10 | 4 | 6 |  | 8 | 2 |  | 9 | 1 |  |
| Neuroendocrine tumor | 3 | 1 | 2 |  | 1 | 2 |  | 1 | 2 |  |
| Sarcoma | 1 | 1 | 0 |  | 1 | 0 |  | 1 | 0 |  |
| **HDAC1 expression** |  |  |  |  |  |  | 0.91 |  |  | 0.45 |
| Negative |  | - | - |  | 47 | 16 |  | 52 | 11 |  |
| Positive |  | - | - |  | 28 | 9 |  | 28 | 9 |  |
| **HDAC2 expression** |  |  |  | 0.91 |  |  |  |  |  |  |
| Negative |  | 47 | 28 |  | - | - |  | 61 | 14 | 0.77 |
| Positive |  | 16 | 9 |  | - | - |  | 19 | 6 |  |
| **HDAC6 expression** |  |  |  | 0.45 |  |  | 0.77 |  |  |  |
| Negative |  | 52 | 28 |  | 61 | 19 |  | - | - |  |
| Positive |  | 11 | 9 |  | 14 | 6 |  | - | - |  |

†Fisher’s exact test, *continuity corrected χ² test

Significant P values were shown in bold

**Supplementary Table 4. Cox-regression analysis of the cohort with inclusion of HDAC expression (n = 100)**

|  | **Univariate HR (95% CI)** | **P value** | **Multivariate HR (95% CI)*** | **P value** |
| --- | --- | --- | --- | --- |
| **CEA level** |  | **0.007** | 1.70 (0.86~3.38) | 0.13 |
| ≤5 ng/ml | 1 |  |  |  |
| > 5 ng/ml | 2.12 (1.23~3.66) |  |  |  |
| **Margin** |  | **< 0.001** | 0.97 (0.29~3.23) | 0.96 |
| R0 | 1 |  |  |  |
| R1/R2 | 2.77 (1.57~4.88) |  |  |  |
| **Liver invasion** |  | **0.002** | 1.75 (0.92~3.35) | 0.09 |
| No | 1 |  |  |  |
| Yes | 2.45 (1.39~4.32) |  |  |  |
|  |  |  |  |  |
| **Nodal status** |  | **< 0.001** | 1.86 (0.35~9.88) | 0.47 |
| N0 | 1 |  |  |  |
| N1~N2 | 3.39 (1.81~6.36) |  |  |  |
| **Stage of metastasis** |  | **< 0.001** | 3.47 (1.19~10.1) | **0.022** |
| M0 | 1 |  |  |  |
| M1 | 4.24 (2.08~8.64) |  |  |  |
|  |  |  |  |  |
| **TNM stage** |  | **< 0.001** | 1.84 (0.38~8.95) | 0.45 |
| Stage 0~IIIA | 1 |  |  |  |
| Stage IIIB-IVB | 3.66 (1.88~7.13) |  |  |  |
| **Differentiation** |  | **0.018** | 2.44 (1.22~5.0) | **0.012** |
| Moderately to well differentiated | 1 |  |  |  |
| Poorly differentiated | 1.98 (1.12~3.49) |  |  |  |
| **Histology** |  | **0.048** | 1.57 (0.96~2.56) | 0.08 |
| Adenocarcinoma | 1 |  |  |  |
| Other types | 1.96 (1.01~3.81) |  |  |  |
| **HDAC1 expression** |  | **0.015** | 2.29 (1.03~5.08) | **0.042** |
| Negative | 1 |  |  |  |
| Positive | 1.95 (1.14~3.33) |  |  |  |
| **HDAC2 expression** |  |  | 1.46 (0.70~3.06) | 0.30 |
| Negative | 1 | **0.036** |  |  |
| Positive | 1.84 (1.04~3.24) |  |  |  |
| **HDAC6 expression** |  | **0.032** | 1.36 (0.58~3.16) | 0.48 |
| Negative | 1 |  |  |  |
| Positive | 1.92 (1.06~3.49) |  |  |  |
|  |  |  |  |  |
| **Age (years)** |  | 0.66 |  |  |
| < 65 | 1 |  |  |  |
| ≥65 | 1.13 (0.66~1.93) |  |  |  |
| **Anti-HBc status** |  | 0.96 |  |  |
| Negative | 1 |  |  |  |
| Positive | 1.02 (0.59~1.74) |  |  |  |
| **Gallstone** |  | 0.64 |  |  |
| No | 1 |  |  |  |
| Yes | 1.14 (0.66~1.95) |  |  |  |
| **CA 19-9 level** |  | 0.20 |  |  |
| ≤37 U/ml | 1 |  |  |  |
| > 37 U/ml | 1.44 (0.83~2.50) |  |  |  |
| **Location** |  |  |  |  |
| Body | 1 |  |  |  |
| Diffused | 2.11 (0.76~5.84) | 0.15 |  |  |
| Neck | 1.96 (0.86~4.47) | 0.11 |  |  |
| Fundus | 1.02 (0.39~2.69) | > 0.90 |  |  |
| **Vascular invasion** |  | 0.88 |  |  |
| No | 1 |  |  |  |
| Yes | 1.06 (0.50~2.25) |  |  |  |
| **Bile duct invasion** |  | 0.08 |  |  |
| No | 1 |  |  |  |
| Yes | 1.60 (0.94~2.74) |  |  |  |
| **Tumor stage** |  | 0.20 |  |  |
| T0-T3 | 1 |  |  |  |
| T4 | 1.53 (0.83~2.81) |  |  |  |

*Covariates with P < 0.05 in univariate analysis were included

Significant P values were shown in bold

**Supplementary Figure 1.** Culture courses of organoids.


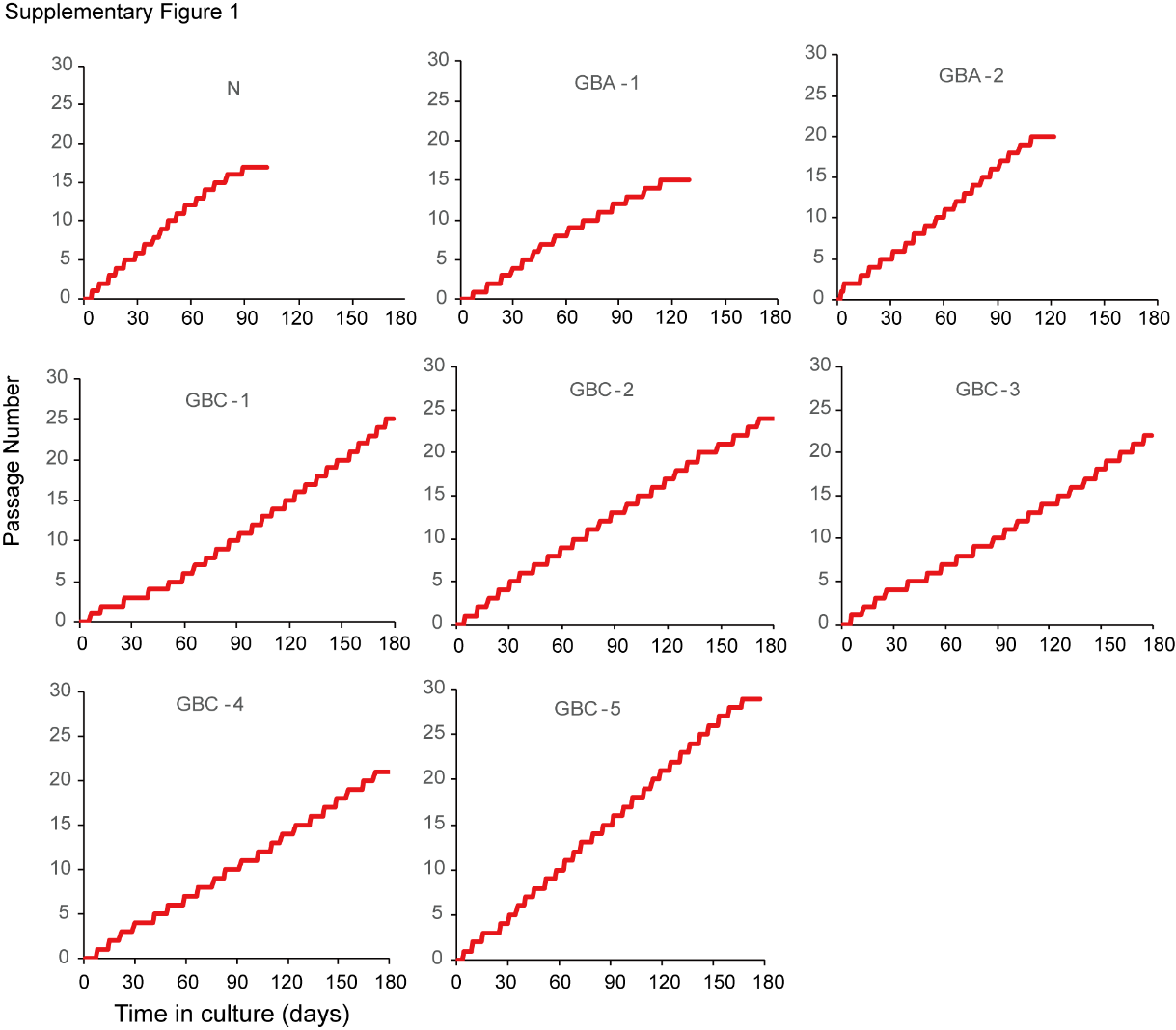


**Supplementary Figure 2.** Heat-map analysis of representative genes in indicated signal pathways for normal and each GBC organoid.


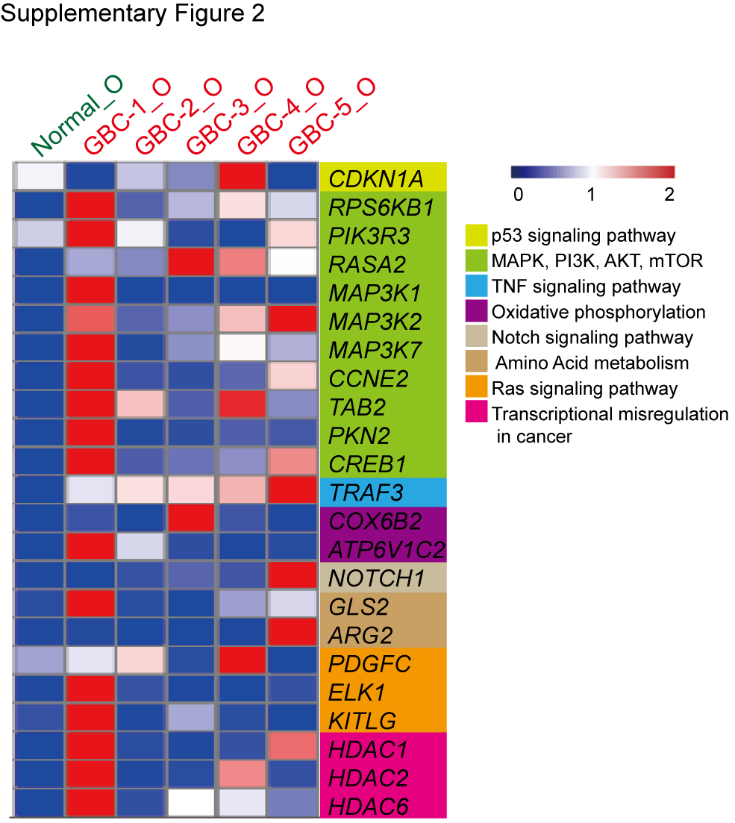


**Supplementary Figure 3.** Heatmap analysis of the differentially expressed genes for GBA-2_T, GBC-4_T and GBC-4_O.


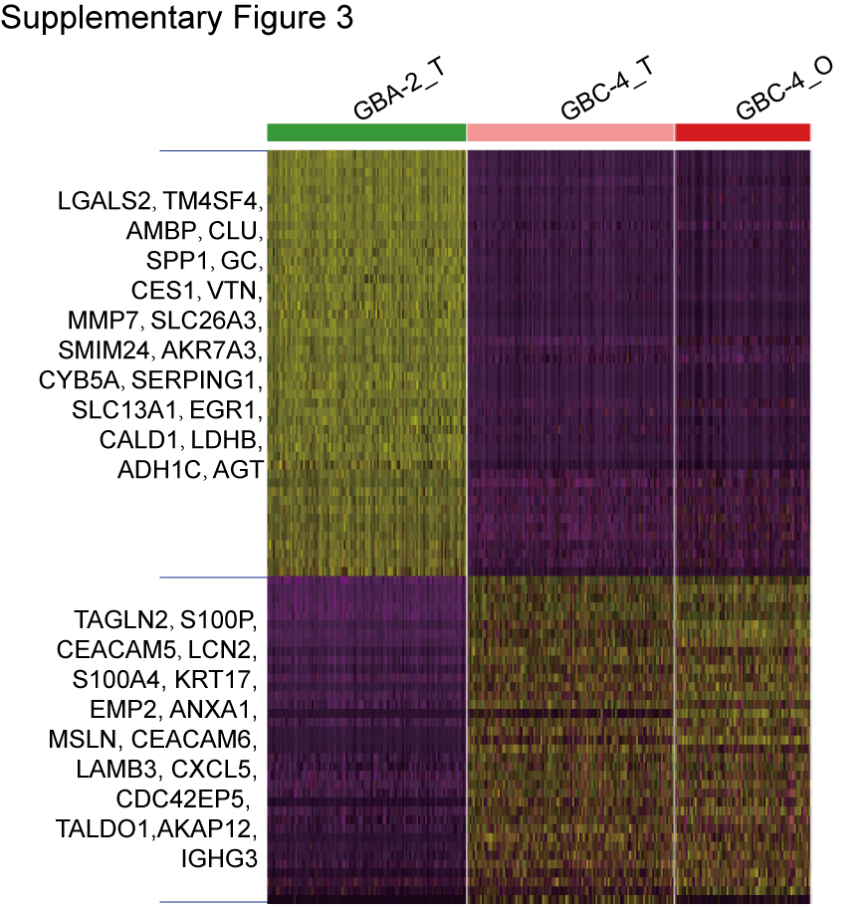


**Supplementary Figure 4.** Heatmap showing transcription factor activity for normal and malignant epithelial cells.


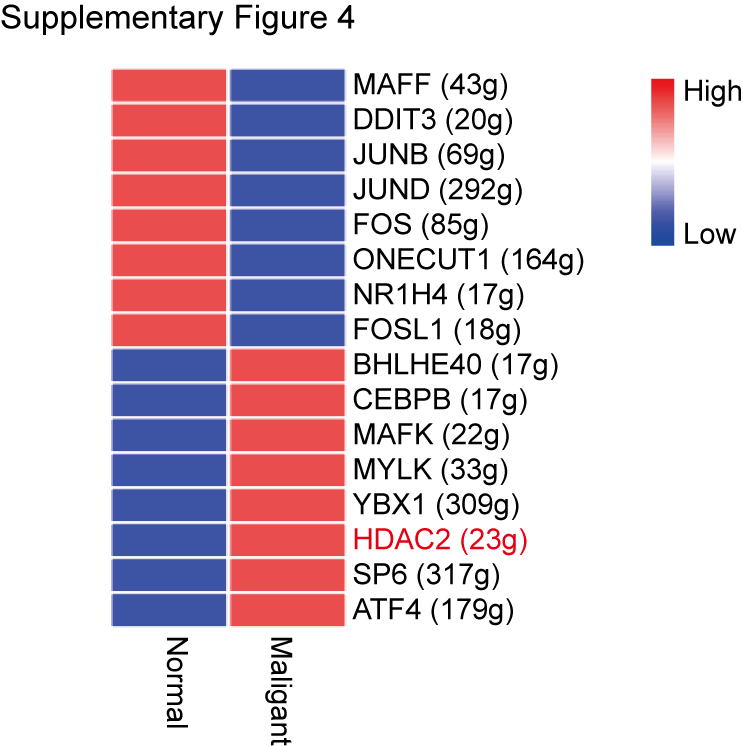

Supplement: Supplementary file 1 — Supporting Information [file CTM2-12-e678-s001.docx]
